# Multi-Scenario Simulation and Trade-Off Analysis of Ecological Service Value in the Manas River Basin Based on Land Use Optimization in China

**DOI:** 10.3390/ijerph19106216

**Published:** 2022-05-20

**Authors:** Yongjun Du, Xiaolong Li, Xinlin He, Xiaoqian Li, Guang Yang, Dongbo Li, Wenhe Xu, Xiang Qiao, Chen Li, Lu Sui

**Affiliations:** 1 College of Water and Architectural Engineering, Shihezi University, Shihezi 832000, China; duyongjun@stu.shzu.edu.cn (Y.D.); lixiaolong409@shzu.edu.cn (X.L.); lixiaoqian@stu.shzu.edu.cn (X.L.); yangguang@shzu.edu.cn (G.Y.); lidongbo@stu.shzu.edu.cn (D.L.); xuwenhe@stu.shzu.edu.cn (W.X.); qiaoxiang@stu.shzu.edu.cn (X.Q.); 2 Key Laboratory of Modern Water-Saving Irrigation of Xinjiang Production and Construction Corps, Shihezi 832000, China; 3 Faculty of Geography, Yunnan Normal University, Kunming 650500, China; 2023130027@user.ynnu.edu.cn; 4 Faculty of Public Administration, Xinjiang Agricultural University, Urumqi 830000, China; 20202110058@stu.shzu.edu.cn

**Keywords:** ecological service value, GMOP–PLUS coupled model, land use changes, trade-offs and synergies, Manas River Basin

## Abstract

Rapid socio-economic development has had a significant impact on land use/cover (LULC) changes, which bring great pressure to the ecological environment. LULC changes affect ecosystem services by altering the structure and function of ecosystems. It is of great significance to reveal the internal relationship between LULC changes and ecosystem service value (ESV) for the protection and restoration of ecological environments. In this study, based on the spatial and temporal evolution of ecological service values in the Manas River basin from 1980 to 2020 and considering ecological and economic benefits, we coupled the gray multi-objective optimization model (GMOP) and patch-generating land-use simulation (PLUS) model (GMOP–PLUS model) to optimize the LULC structure under three scenarios (a natural development scenario, ND; ecological priority development scenario, (EPD); and balanced ecological and economic development scenario, EED) in 2030, and analyzed the trade-offs and synergies in the relationships among the four services. We found that from 1980 to 2020, farmland and construction land expanded 2017.90 km^2^ and 254.27 km^2^, respectively, whereas the areas of grassland and unused land decreased by 1617.38 km^2^ and 755.86 km^2^, respectively. By 2030, the trend of LULC changes will be stable under the ND scenario, the area of ecological land will increase by 327.42 km^2^ under the EPD scenario, and the area of construction land will increase most under the EED scenario, reaching 65.01 km^2^. From 1980 to 2020, the ESV exhibited an upward trend in the basin. In 2030, the ESV will increase by 7.18%, 6.54%, and 6.04% under the EPD, EED, and ND scenarios, respectively. The clustering of the four services is obvious in the desert area and around the water system with “low–low synergy” and “high–high synergy”; the plain area and mountainous area are mainly “high–low trade-off” and “low–high trade-off” relationships. This paper provides a scientific reference for coordinating economic development and ecological protection in the basin. It also provides a new technical approach to address the planning of land resources in the basin.

## 1. Introduction

Ecosystem services are the products and services provided by ecosystem structures, functions, and processes related to human productivity and life which allow humans to obtain direct or indirect benefits from ecosystems [1]. As an important hub of human production and activities, the ecosystem provides stable and sustainable ecosystem services for the realization of social well-being [2,3]. According to the United Nations Millennium Ecosystem Assessment, about 60% of the global ecosystem services have been degraded in the past 50 years, mostly due to human activities that have damaged the value and functions of ecosystem services [4]. Numerous factors affect ecosystem services, especially changes in land use/cover (LULC), socio-economic factors, and the natural environment; these factors can all change the ecosystem service value (ESV) by affecting the ecological development process [5]. In particular, the high-intensity use of land can significantly change the LULC pattern, which profoundly affects the service capacity of the ecosystem; the unbalanced development of the LULC structure severely reduces the ESV [6,7]. Rapid economic growth and the large population of China have imposed great pressure on the ecological environment [8,9,10];thus, it is necessary to determine how to balance the economic and social benefits brought by social development with the ecological benefits obtained from sustainable development. Therefore, the relationships between LULC changes and the ESV should be explored in order to facilitate the healthy development of the ecosystem.

In order to improve ecological environment quality, scholars around the world improve and optimize LULC structure by studying changes in ESV with the goal of enhancing ecosystem service capacity [11,12]. In China, the important concept of “ Mountains, rivers, forests, fields, lakes and grasses are a community of life” was put forward from the perspective of land’s natural carrying capacity and ecological coordination, and various schemes and strategies have been implemented to effectively coordinate the allocation of land resources and ensure balanced regional development [13]. Therefore, optimizing the structure of LULC is crucial for the quality of the regional ecological environment and its service capacity [14,15]. However, few studies have conducted research and produced reference data regarding optimization of the regional LULC structure, especially LULC structure optimization based on ESV.

LULC changes can have a significant impact on the ESV [16,17], but it is necessary to predict future LULC changes to understand the potential relationship between LULC and ESV [18]. Combining LULC change research with the ESV under future scenarios can provide a scientific basis for the rational planning of land resources [19,20,21]. Previous research into LULC changes and the ESV can be divided into the following three categories. (1) Most studies investigated the relationships between changes in LULC and the ESV under historical development conditions, but they did not deeply explore the two relationships under different scenarios in the future [22,23]. (2) Some studies used algorithms to optimize the LULC structure and conducted quantitative research into the ESV under multiple scenarios in the future; however, they could not express the spatial dynamic changes of LULC and ESV, rendering the research results incomplete [24,25]. (3) Other studies used Markov, cellular automata (CA), and CLUE-S models to simulate the spatial changes in LULC, but they did not optimize the LULC structures, thereby resulting in differences between the simulation results and actual changes in LULC development [26,27,28]. In general, previous studies generally focused on simulating and optimizing a single aspect of the quantitative structure or spatial layout of LULC, and the settings used in simulation scenarios were also highly subjective. In addition, the simulation methods used, such as cellular automata (CA) or CLUE-S, are bottom-up methods to assign different LULC types to suitable locations. These models rely on their own transformation rules and lack flexible patch-based strategies to model patch growth across multiple natural LULC types at a fine-scale resolution. Therefore, the land use structure cannot be optimized well. There is little research on LULC optimization from the perspective of improving the comprehensive benefit of LULC, especially on the collaborative optimization of LULC quantity, spatial layout, and benefit by using multi-objective optimization scenarios combined with models.

In order to solve the problems with previous research, it is necessary to consider whether the simulated LULC pattern can meet the needs of future regional expansion and urban population growth, as well as the requirements of regional sustainable development. The LULC structure should be suitable for the natural, social, and economic conditions in a region to ensure that a long-term balance is achieved between economic development, environmental protection, and the efficient use of resources, thereby ultimately leading to the ecological security of a region and its sustainable development [29]. Therefore, we developed a coupled model based on the gray multi-objective optimization (GMOP) and patch-generating land-use simulation (PLUS) models [15,30], combining the advantages of the two models in terms of quantity and space optimization. We set the objective function from the perspective of ecological and economic maximization, set constraints from the three aspects of society, economy, and ecology, and set different scenarios (natural development scenario, ND; ecological priority development scenario, EPD; and balanced ecological and economic development scenario, EED) for future regional development. The GMOP–PLUS coupling model is used to realize the optimization of LULC quantity and space under different scenarios, so as to simulate the LULC changes under different scenarios in the future. We used the GMOP–PLUS coupling model to study the optimization of LULC structure to make up for the single aspect of land use quantity or space optimization and to enhance the scientific setting of future scenarios. In addition, the accuracy of spatial optimization of LULC is increased, as the coupling model is further improved in spatial transformation rules. GMOP is obtained by the combination and development of grey programming and multi-objective programming; this model can solve various uncertainties of objective functions and constraints in actual LULC as well as solving the multi-objective problems in the process of LULC structure optimization. The model has four main parts: the decision variable selection, objective function setting, gray constraint determination, and solution method selection components [31,32]. The PLUS model exploits the advantages and addresses the shortcomings of the transformational analysis strategy and pattern analysis strategy for LULC simulation [27,28]. This model is more focused on the relationships between LULC changes and various drivers, and it solves the problem of insufficient capacity for large-scale regional patch evolution, thereby improving the precision and accuracy of LULC simulations [30,33].

The Manas River Basin is located in the arid zone of northwest China, and it has a distinct natural environment characterized by a mountain–oasis–desert complex ecosystem (MODS). This ecological environment is highly fragile and sensitive due to unique geographical and climatic conditions. Changes in the natural environment and human activities have influenced the development of the basin. In the present study, we combined the GMOP and PLUS models to study the changes in LULC in the Manas River Basin, as well as exploring the relationships between changes in the ESV and LULC patterns. The objectives of this study were: (1) to analyze the temporal and spatial changes in LULC and ESV in the Manas River Basin from 1980 to 2020; (2) to predict the changes in LULC in the Manas River Basin in 2030 under three scenarios (ND, EPD, and EED) using the GMOP–PLUS coupled model; and (3) to measure the trade-offs and changes in the ESV in the Manas River Basin in 2030 under different scenarios. The optimized method proposed in this study is useful for exploring changes in the LULC and ESV in basins, and it may provide a scientific basis and reference for the rational planning of basin land resources and ecological environmental protection.

## 2. Material and Methods

### 2.1. Overview of the Study Area

**Figure 1:**
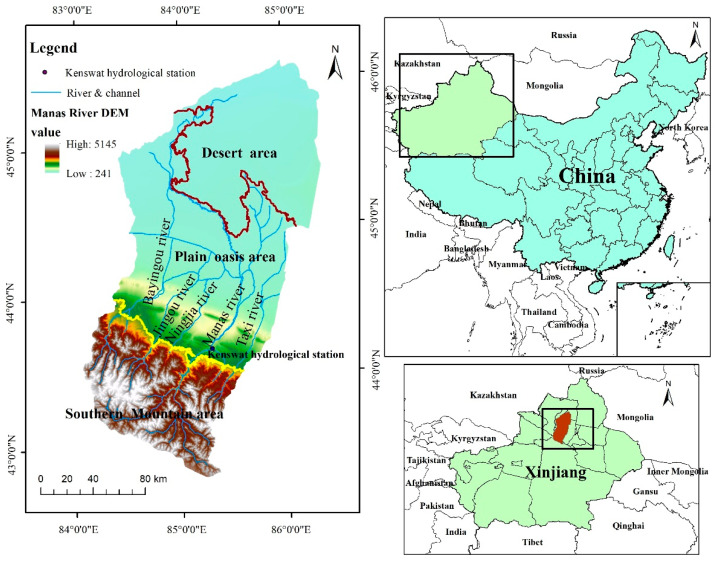
Location and overview of the study area.

The Manas River Basin is located in the hinterland of the Eurasian continent, and it is a typical arid zone in northwest China. The basin is located at the northern foot of the Tianshan Mountains in Xinjiang and at the southern edge of the Junggar Basin, with approximate geographical coordinates of 43°27′–45°21′ N and 85°01′–86°32′ E [34]. The administrative divisions included in the basin mainly comprise 10 cities and counties, including Shihezi City, Manas County, Shawan County, and Bursek Mongol Autonomous County. The basin has an area of about 3.4 × 10^4^ km^2^ and it contains six rivers, including the Manas River, Bayingou River, and Jingou River [35]. The Manas River Basin has a typical temperate continental arid climate, which is characterized by aridity, low rainfall, and high-intensity evaporation, with an average annual temperature of around 5.9 °C, average annual precipitation of 100–200 mm, and average annual evaporation of 1500–2100 mm. The topography of the basin is characterized as high in the east and low in the west and high in the south and low in the north, and the altitude varies significantly, from 241 m to 5242 m. The basin is divided from south to north into the southern mountainous region, oasis plain region, and desert region, where the southern mountainous region is the water production area, the plain region is the agricultural production area, and the desert region is the water dissipation area (Figure 1).

### 2.2. Data Ssources

LULC, average annual temperature, annual rainfall, socio-economic, and water system data were acquired from the Resource and Environmental Science and Data Centre (http://www.resdc.cn). LULC data were classified by the Chinese Academy of Sciences as farmland, woodland, grassland, water area, construction land, and unused land, with an accuracy of at least 94.3% [36]. Road data were obtained from Open Street Map (http://www.openstreetmap.org) and elevation data from Geospatial Data Cloud (http://www.gscloud.cn/search). Grain crop area and production data were obtained from the Xinjiang Production and Construction Corps Statistical Yearbook, Xinjiang Statistical Yearbook, and China Grain Yearbook.

### 2.3. Research Methods

**Figure 2:**
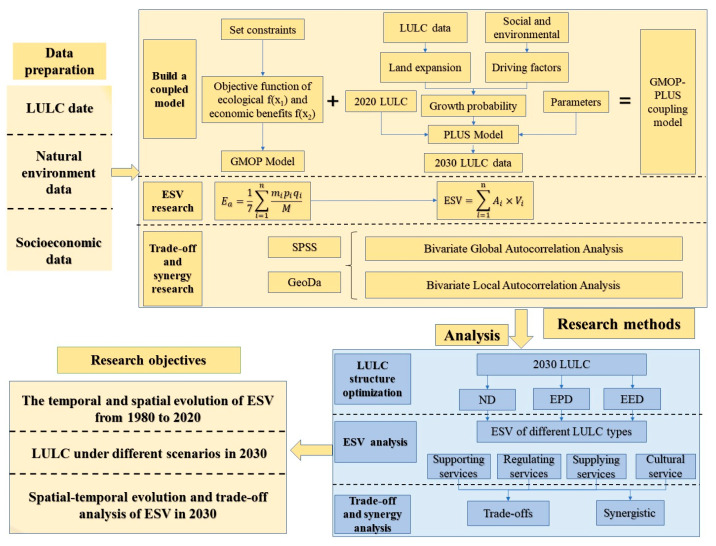
The research framework of this paper. LULC, Land use/cover; ESV, Ecosystem service value; ND, Natural development scenarios; EPD, Ecological priority development Scenario; EED, Balanced ecological and economic development; GMOP, Gray multi-objective optimization; PLUS, Patch-generating land-use simulation.

Based on the spatial-temporal evolution characteristics of ESV in the Manas River Basin, this study constructed a GMOP–PLUS coupling model to optimize LULC structure under three scenarios in 2030 and calculated and analyzed the spatial correlation of ESV under the three scenarios. The research framework is shown in Figure 2.

#### 2.3.1. GMOP–PLUS Model

**Figure 3:**
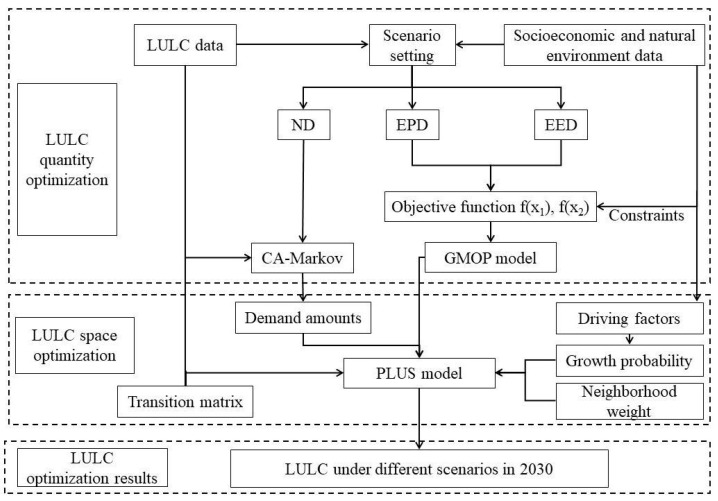
Coupling flow chart of GMOP–PLUS model.

The construction and application of the GMOP–PLUS model involved three steps (Figure 3). First, we combined the LULC changes under historical conditions in the study area and the constraints on the social economy and natural environment. Three scenarios, ND, EPD, and EED scenarios, were set for the future, and CA-Markov and GMOP models were used to predict the LULC quantitative data under these three scenarios in 2030. Second, the spatial distributions of LULC under the three scenarios were simulated using the PLUS spatial optimization model. Finally, by integrating quantitative optimization and spatial optimization, we obtained the optimal structure of LULC within different scenarios in the study area in 2030.

##### Optimization of the LULC Structure Using the GMOP Model

GMOP was developed based on the gray theory system and GM(1,1) model combined with multi-objective planning [32]. The model is multi-objective and dynamic, and it uses multiple social, economic, and ecological constraints to solve the LULC optimization problem. Using the GMOP model and Lingo 11.0 software, we predicted the changes in the area of each LULC type in 2030 to provide the best LULC structure optimization plan for decision makers. The GMOP model is constructed as follows:

(1) $$f\left(X\right)=\overset{m}{\underset{j}{\sum }}c_{j}X_{j}\;\to \;\max\;\left(\min\right)$$

(2) $$\overset{m}{\underset{j=1}{\sum }}a_{ij}\;\geq \;\left(\leq \right)\;b_{i}\;\left(i=1,\;2,\ldots \;m\right)$$

(3) $$X_{j}\;\geq \;0\;\left(j=1,\;2,\ldots \;n\right)$$

where *f(X)* is the objective function and it is determined according to different benefit goals, *X_j_* is the decision variable, which refers to the LULC type of the study area in the LULC optimization research, *c_j_* is the benefit coefficient, which generally refers to the benefit contribution weight of each decision variable, *a_ij_* is a constraint condition, and LULC is affected by various economic, social, and natural factors. In actual operation, model constraints are formulated according to the actual situation of the region itself. *b_i_* is a constraint constant, which can usually be predicted, or is a planning constraint and a government’s macro policy constraint.

(a) Multi-scenario settings

Natural development scenario (ND): Based on the changing trend of regional LULC, the future LULC pattern was linearly predicted. This scenario assumes that each LULC type is not restricted by any planning policy on LULC changes, and only develops linearly according to its historical evolution trajectory, which is an ideal LULC development scenario. According to the characteristics of LULC changes and the probability matrix of LULC in the Manas River Basin from 2015 to 2020, and based on the LULC data in 2020, we used the Markov-chain module in the PLUS model to predict the spatial structure of LULC in the study area in 2030.

Ecological priority development scenario (EPD): The development of society and economy has caused serious actual and potential harm to the ecological environment of the Manas River Basin. To promote the high-quality development of the basin, environmental protection must be placed in a prominent position. This scenario ensures the priority of basin ecological development by setting ESV maximization so that each LULC type can provide maximum ecological benefits. Under the EPD scenario, the GMOP objective function was set to reach the maximum value for the basin ESV. The objective function is:

(4) $$f_{1}\left(X\right)=max\overset{n}{\underset{j=1}{\sum }}c_{j}X_{j}$$

where *f*_1_*(X)* is *ESV* (unit: 10^4^ CNY/ha, CNY = Chinese Yuan), *X_j_* represents the decision variable for LULC type *j* (*X*_1–6_ represent farmland, woodland, grassland, water area, construction land, and unused land, respectively), and *c_j_* is the *ESV* per unit area, where the *c_j_* (unit: 10^4^ CNY/ha) values for *X*_1–6_ are 0.5427, 2.4500, 1.1617, 6.0942, 0.0011, and 0.0577, respectively.

Balanced ecological and economic development scenario (EED): Economic development and ecological protection are not mutually exclusive; they can promote and complement each other. The mode of coordinated development of ecology and economy is to organically combine natural ecology with social economy to achieve healthy, sustainable, and stable development of humans and nature. This scenario aims to build a long and harmonious man–land relationship by maximizing economic and ecological benefits. Considering overall ecological and economic development, the objective function was set for GMOP so that the ESV and economic benefits reached their maximum values in the Manas River Basin. The objective function is:

(5) $$Max\;\left\{f_{1}\left(X\right)\;f_{2}\left(X\right)\right\}$$

(6) $$f_{2}\left(X\right)=max\overset{n}{\underset{j=1}{\sum }}d_{j}X_{j}$$

where *f*_2_*(X)* is the economic benefit of the study area (unit: 10^4^ CNY/ha), *d_j_* is the economic benefit coefficient per unit area, and the weight vectors for the land economic benefits of *X*_1–6_ in the Manas River Basin were determined using the analytic hierarchy process [37] as 0.3239, 0.0555, 0.1477, 0.0928, 1.7539, and 0.0001, respectively. The gray GM(1,1) model was used to predict the economic benefit of LULC per unit area in the Manas River Basin in 2030, and the economic benefit coefficients of *X*_1–6_ in 2030 were calculated as 3.5365, 0.6060, 1.6127, 1.0132, 19.1502, and 0.0011, respectively.

(b) Constraint setting

The aim of optimal LULC structure allocation is to achieve sustainable regional development. Therefore, the LULC structure should be optimized according to the future development guidelines and policies for the Manas River Basin.

(1) Total land

(7) $$X_{1}+X_{2\;}+X_{3}+X_{4}+X_{5}+X_{6}=3405150\;\left(ha\right)$$

(2) Population

The total population of construction land should be kept within the target annual population range for the study area:

(8) $$M\times X_{5}\;\leq \;P$$

where *M* represents the average population density of construction land and *P* represents the total population. Using the gray GM(1,1) model to predict the population density and total population of construction land in 2030, *M* was determined as 13.5825 and *P* as 1012234.

(3) Farmland

According to the requirements of the Xinjiang Uyghur Autonomous Region and Xinjiang Construction Corps to “withdraw land and reduce water,” the farmland area and irrigation water consumption should be reduced to replenish ecological water while also protecting the basic farmland area in each region to ensure development of the agricultural economy. The arable farmland area should be smaller than the current arable land area, thus:

(9) $$732,848.78\;\leq \;X_{1}\;\leq \;780,448.78\;\left(ha\right)$$

(4) Woodland

Due to the implementation of “returning farmland to woodland” and to ensure the protection of forests by ecological construction in China, the woodland area should not be smaller than the forest area in the Manas River Basin in 2020, thus:

(10) $$X_{2}\;\geq \;45551.59\;\left(ha\right)$$

(5) Grassland

The total area of grassland decreased each year from 1980 to 2020; it was mainly transformed into cultivated land. According to the “Overall Plan for Land Use in Xinjiang Uygur Autonomous Region (2006–2020)” and the policy of “Returning Farmland to Grassland”, in order to accelerate the ecological replacement of grassland and strengthen the ecological protection of grassland, the grassland area should not be smaller than the current area. Combined with the development of the watershed, the grassland area should not exceed the pre-cultivation area in 1980, and thus:

(11) $$966377.47\;\leq \;X_{3}\;\leq \;1128115.56\;\left(ha\right)$$

(6) Water area

With the implementation of the policy of “land retreat and water reduction”, the water used for agricultural production is reduced to compensate for ecological water consumption. Based on the Markov chain, the water area in the natural development scenario is calculated to be 129512.47 ha, which we set as the lower limit. From 2015 to 2020, the water area increased at the greatest rate; based on this growth rate, the basin area of the Manas River basin was calculated as 131892.00 ha in 2030. We set this as the upper limit, thus:

(12) $$129512.55\;\leq \;X_{4}\;\leq \;131892.00\;\left(ha\right)$$

(7) Construction land

According to the Xinjiang Town System Plan (2012–2030) and General Land Use Plan for the Xinjiang Uygur Autonomous Region (2006–2020), considering both urban development and economic development, the area of construction land in the Manas River Basin in 2030 should not be smaller than the area calculated for the ND scenario, and thus:

(13) $$X_{5}\;\geq \;56888.94\;\left(ha\right)$$

(8) Unused land

In order to improve the social economy, the value of unused land should be explored as much as possible. Due to the expansion of construction land and increased ecological protection, the unused land tended to decrease by 1.24% over the years. The unused land area in 2030 was calculated as 1,428,217.15 ha, which was set as the lower limit, and the current unused land area was set as the upper limit, thus:

(14) $$1428217.15\;\leq \;X_{6}\;\leq \;1453609.70\;\left(ha\right)$$

(9) ESV

The value of ecosystem services in the Manas River Basin is primarily due to provisioning, regulating, and supporting services, all of which should be valued above the current ESV, and thus:

(15) $$0.0673\times X_{1}+0.3588\times X_{2\;}+0.0897\times X_{3}+2.0116\times X_{4}+0.0034\times X_{6}\;\geq \;371430\;\left(CNY\right)$$

(16) $$0.0011\times X_{1}+0.4373\times X_{2}+0.1514\times X_{3}+0.0964\times X_{4}+0.0043\;X_{6}\;\geq \;180450\;\left(CNY\right)$$

(17) $$0.0235\times X_{1}+0.3655\times X_{2}+0.1503\times X_{3}+0.2803\times X_{4}+0.0381\times X_{6}\;\geq \;26505\;\left(CNY\right)$$

(10) Model self-constraint

Each constraint variable in the model is non-negative.

(18) $$X_{i}\;\geq \;0,\;i=1,2,3\ldots ,6$$

##### Optimization of the LULC Spatial Structure Using the PLUS Model

The PLUS model is an improved patch land use simulation model based on the FLUS model, which can more accurately reveal the non-linear intrinsic relationships behind patch-level changes in LULC [15]. The model primarily couples the Land Expansion Analysis Strategy (LEAS) mining framework with a multi-class random patch seed-based CA model (CARS). The model also uses the Random Forest algorithm to mine the factors related to each type of LULC expansion and various drivers in order to determine the probabilities of different types of LULC development and the contributions of these drivers to each type of land expansion. In addition, the model combines the advantages of the existing Transformation Analysis Strategy (TAS) and Pattern Analysis Strategy (PAS) in the commonly used CA models, as well as retaining the model’s ability to analyze mechanisms related to LULC changes over a certain period of time. Stochastic seed generation and threshold-decreasing mechanisms are applied to better simulate changes at the patch level for multiple types of LULC and increase the accuracy of the simulation results [15]. In conclusion, the PLUS model can comprehensively consider the underlying mechanisms related to LULC changes and improve the model’s ability to simulate the real LULC. The main calculation modules are described as follows:

(1) Suitability probability calculation

Under the PLUS model land expansion analysis strategy module, the random sampling mechanism is used to reduce the model calculation amount; at the same time, the random forest algorithm is used to mine the transition law of LULC transfer and obtain the development probability of each LULC type:

(19) $$P_{i,k}^{d}\left(x\right)=\frac{\sum_{n=1}^{M}I\left(h_{n}\left(x\right)=d\right)}{M}$$

where, the value of *d* is 0 or 1 (if the value is 1, other LULC types can be converted to land type *K*; if the value is 0, other LULC types cannot be converted to land type *k*), *X* is a vector consisting of drive factors, *h_n_(x)* is the type of LULC prediction calculated when the decision tree is *n*, *I* (·) is the indicator function of the decision tree, and $P_{i,k}^{d}\left(x\right)$ is the probability of growth of k-class LULC type at spatial unit i, under the condition that d is 0 or 1 [38].

(2) Neighborhood weight setting

Neighborhood weight is mainly used to reflect the degree of difficulty of conversion between different LULC types [30], and the formula is as follows:

(20) $$\Omega_{i,k}^{t}=\frac{con\left(c_{i}^{t-1}=k\right)}{n\times n-1}\times w_{k}$$

where *w_k_* is the neighborhood weight parameter of the land type *k*, between [0, 1]; *n ×* *n* is the cell unit, this study sets *n* = 3; con(c_i_^t^^−1^=k) represents the total number of grid cells occupied by LULC type *k* at the last iteration within the *n × n* window; and $\Omega_{i,k}^{t}$ is the neighborhood weight of class *k* at space unit *i* at time *t*.

(3) Adaptive inertial competition mechanism

Adaptive inertia can be adaptively adjusted in the iterative process according to the difference between the expected demand of land quantity and the existing situation so that the quantity of LULC develops towards the expected target, and the formula is as follows:

(21) $$D_{k}^{t}=\{\begin{array}{c} D_{k}^{t-1}\;\;\;\;\;\;\;\;\;\;\;\;\;\;\;\;if\;\left|G_{k}^{t-1}\right|\;\leq \;\left|G_{k}^{t-2}\right|\\ D_{k}^{t-1}\times \frac{G_{k}^{t-2}}{G_{k}^{t-1}}\;\;if\;0>G_{k}^{t-2}>G_{k}^{t-1}\\ D_{k}^{t-1}\times \frac{G_{k}^{t-1}}{G_{k}^{t-2}}\;if\;G_{k}^{t-1}>G_{k}^{t-2}>0 \end{array}$$

where $D_{k}^{t}$ is the inertia coefficient of the land type *k* at time *t* and $G_{k}^{t-1}$ and $G_{k}^{t-2}$ are the difference between the current quantity and future demand of land use type *k* at iterations *t* − 1 and *t* − 2, respectively [39].

(4) Random patch generation parameter settings

The random patch generation mechanism can better simulate the natural growth of various types of land in the process of real LULC changes. In order to control the generation of multi-type LULC patches, the model proposes a threshold drop rule using a competitive process to limit the spontaneous growth of each LULC type; the formula is as follows [30]:

(22) $$OP_{i,k}^{d=1,t}=P_{i,k}^{d}\times \Omega_{i,k}^{t}\times D_{k}^{t}$$

(23) $$TP_{i,k}^{d=1,t}=\{\begin{array}{c} P_{i,k}^{d=1}\times \left(r\times u_{k}\right)\times D_{k}^{t}\;if\;\Omega_{i,k}^{t}=0,r<P_{i,k}^{d=1}\\ P_{i,k}^{d=1}\times \Omega_{i,k}^{t}\times D_{k}^{t} \end{array}$$

where $OP_{i,k}^{d=1,t}$ is the comprehensive probability of the transformation of space unit *i* to LULC type *k* at time *t*, $P_{i,k}^{d=1}$ is the suitability probability of LULC type developing to *k* at space unit *i*, $\Omega_{i,k}^{d}$ is the neighborhood weight of class *k* at space unit *i* at time *t*, $TP_{i,k}^{d=1,t}$ is the adaptive drive factor, $TP_{i,k}^{d=1,t}$ is the comprehensive probability of the transformation of space unit *i* to LULC type *k* at time *t* after random patch generation, *r* is a random value between 0–1, and *u_k_* is the new LULC patch generation threshold.

(5) Transition matrix and final land development probability calculation

The transition matrix is used to define whether the conversion between land types is possible, which can effectively constrain the unreasonable conversion between land types. In addition, the attenuation coefficient of patch generation threshold is set to constrain the spontaneous growth process of LULC type and determine the final LULC method [30]. The formula is as follows:

(24) $$if\;\overset{N}{\underset{k=1}{\sum }}\left|G_{c}^{t-1}\right|-\overset{N}{\underset{k=1}{\sum }}\left|G_{c}^{t}\right|<Step\;Then\;l=l+1$$

(25) $$\{\begin{array}{c} P_{i,c}^{d=1}>\tau \;and\;TM_{k,c\;}=1\\ P_{i,c}^{d=1}\;\leq \;\tau \;or\;TM_{k,c}=0 \end{array}\;\tau =\delta^{l}\times r1$$

where *Step* is the step size of the PLUS model to approximate the LULC demand, *δ* is the decay factor of decreasing threshold *τ* between 0 and 1, *r*1 is a normal distribution with mean 1, and *TM_k,c_* is the transition matrix that defines whether LULC type *k* is allowed to convert to type *c* (a value of 1 means the transition is allowed and a value of 0 means the transition is restricted).

(a) PLUS model input settings

In this study, considering the actual situation in the study area and the availability of data for relevant factors in the basin, we calculated suitable probabilities for different types of LULC using the eight factors that drive changes in the LULC. These drivers, comprising natural factors (temperature, precipitation, elevation, and slope), socio-economic factors (GDP density and population density), and accessibility factors (highways, railways, city-level roads, and water systems) were rasterized and unified in a projection coordinate system with a uniform spatial resolution of 100 m to ensure efficient computations. The neighborhood weight parameter reflects the ability of different LULC types to expand under the action of drivers, with a threshold value between 0 and 1, where higher values indicate a greater ability to expand for a type of land [40]. The characteristic historical changes in LULC in the study area [3] were used to set values for farmland (0.4084), woodland (0.4851), grassland (0.4671), water area (1), construction land (0.5501), and unused land (0.1).

(b) Accuracy verification

In order to verify the simulation accuracy of the PLUS model, this study uses the LULC data in 2015 and 2020, combines parameters such as development probabilities of different LULC types and neighborhood weights, predicts the scale of various types of LULC in the study area in 2020 using the Markov-chain module in the PLUS model, uses this as a basis to simulate the LULC changes under comprehensive spatial optimization to obtain the LULC data of the Manas River Basin in 2020, compares this result with the actual value in 2020, and then calculates the total simulation accuracy and the Kappa coefficient. The value of overall accuracy and Kappa coefficient is closer to 1, which indicates better simulation accuracy; when the Kappa coefficient is greater than 0.8, it means that the model simulation accuracy is satisfactory in terms of statistical significance [41,42]. Through calculation, it was found that the overall accuracy of this verification is 97.46%, the Kappa coefficient is 0.9628, and the experimental simulation accuracy has reached a high level, indicating that the PLUS model has good applicability in this study.

#### 2.3.2. ESV

The ESV was investigated using the value equivalent approach. The “ESV per unit area of terrestrial ecosystems in China” and the “ESV per unit area base equivalence table” were derived from the study by Xie Gaodi et al. [43] and revised based on food prices and biomass.

##### Revision Based on Food Prices

Considering the differences in social and economic conditions, we first corrected the economic value of grain crops per unit area of farmland based on the average price of grain crops in the Manas River Basin in 2018 and the yield per unit of grain crops. According to the study by Gaodi et al. [44], under conditions with no labor input, the economic value of the ecosystem in the study area is 1/7 of the economic value of the grain yield per unit area of farmland, and thus the revision method is as follows:

(26) $$E_{a}=\frac{1}{7}\overset{n}{\underset{i=1}{\sum }}\frac{m_{i}p_{i}q_{i}}{M}$$

where *E_a_* is the economic value of production from a farmland ecosystem per unit area, CNY/ha; *n* represents the number of crop species; *i* is the type of crop; *m_i_* is the planted area of crops, ha; *p_i_* is the national average unit price for a crop, CNY/kg; *q_i_* is the yield per unit area of the crop, kg/ha; and *M* represents the total area of crop cultivation, ha.

##### Biomass-Based Revision

Considering the spatial and temporal variability in ecosystem conditions, the biomass factors for farmland ecosystems in various provinces of China reported by Xie Gaodi et al. [45] were used to determine a biomass factor of 0.58 for farmland ecosystems in the study area. In addition, the biomass factor of ecosystem service value was revised based on the “Ecosystem service value per unit area of China’s terrestrial ecosystem”, “Ecosystem service equivalent value per unit area” [43], and the actual situation of the Manas River Basin. The economic value of grain yield per unit area of farmland in the Manas River Basin can be determined by the sown area and yield of main grain crops in the research area and the average grain price of Xinjiang. According to formula (26), the economic value of annual grain yield of farmland in the Manas River Basin can be calculated as 1121.29 CNY/ha. According to the revised biomass factor and the calculated annual economic value of cultivated land, the coefficient of ESV per unit area was calculated (Table 1).

##### ESV Calculation

ESV was calculated using formula (27) and the coefficient of ESV per unit area. The formula used for the calculation is as follows [46]:

(27) $$\mathrm{ESV}\;=\overset{6}{\underset{i=1}{\sum }}A_{i}\times V_{i}$$

where ESV is the ESV for the LULC type, CNY; *A_i_* is the area of this type of land, ha; and *V_i_* is the equivalent value of ESV, CNY/ha.

In order to analyze the spatial difference of ESV caused by LULC changes in the basin, the grid was selected as the evaluation unit. Referring to previous research results and combined with the size of the research area, the results show that the commonly used grid analysis units mainly include 500 m × 500 m [47], 1 km × 1 km [48], 2 km × 2 km [49], and 3 km × 3 km [50]. Considering the influence of study area on the selection of grid units, this study compared ESV under different grid units and found that the grid size of 2 km × 2 km could highlight the spatial difference of ESV in the Manas River Basin. Therefore, a 2 km × 2 km grid was selected as the evaluation unit to characterize the spatial differentiation of ESV, and the natural breakpoint method was used to divide it into seven levels (I–VII) from low to high.

##### Sensitivity Analysis of ESV

The accuracy of the assessed ESV results was tested via sensitivity analysis. Many previous studies of economics used the elasticity coefficient to calculate the sensitivity index and to test the calculated ESV results in order to determine the dependence of the ESV on the ESV coefficient [51]. We used the coefficient of sensitivity (CS) to test the accuracy of the ESV results. The ESV coefficient was adjusted for different LULC types in the Manas River Basin (±) 50% to calculate the change in the total ESV in the basin. If CS > 1, the ESV is elastic to the value of the coefficient for ecological services; if CS < 1, the ESV lacks elasticity. The results indicated that the ESV coefficient was suitable for the study area. The accuracy of the value coefficient is more critical when the CS value is greater [46,52]. 

#### 2.3.3. Spatial Autocorrelation Analysis

This paper uses spatial autocorrelation analysis to characterize the clustering and divergence of ESV. Spatial autocorrelation is divided into two categories: global autocorrelation and local autocorrelation [53]. Ansenlin [54] proposed a bivariate spatial autocorrelation for measuring the level of spatial association between two attribute variables. In this study, spatial autocorrelation analysis was conducted using SPSS and GeoDa software.

## 3. Results

### 3.1. Spatial and Temporal Changes in LULC in the Manas River Basin

**Figure 4:**
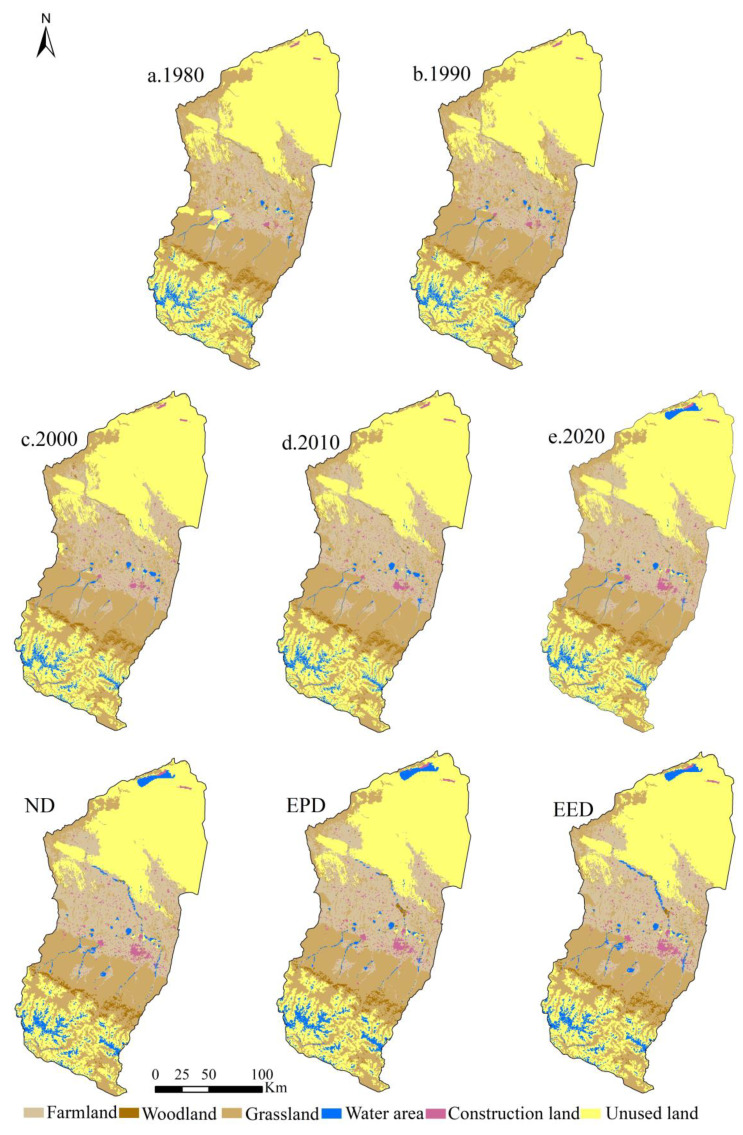
Spatial distribution characteristics of LULC in the Manas River Basin.

**Figure 5:**
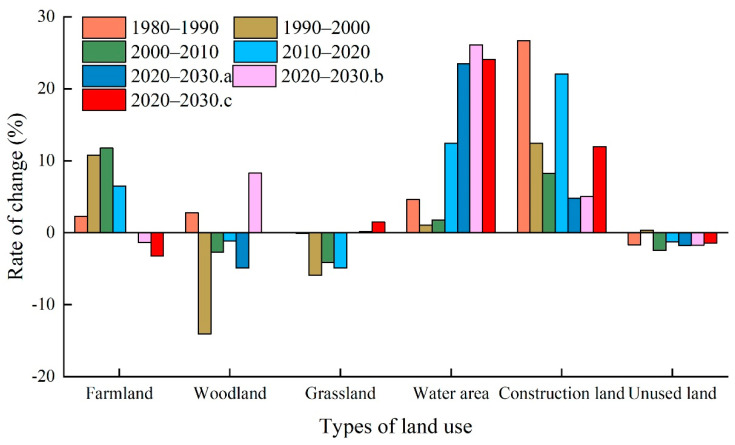
Changes in LULC types in the study area from 1980 to 2030.(Note: a, b, and c indicate ND, EPD, and EED scenarios, respectively).

As can be seen from Figure 4, the spatial distribution of farmland, grassland, and unused land in the Manas River basin is relatively widespread, with farmland and grassland mainly located in the oasis plains and unused land mainly in the southern mountainous and desert areas. Due to the impact of the natural environment, the area occupied by water area and woodland is relatively small, where woodland was mainly distributed in the northern part of the southern mountainous area and water areas were mainly distributed in the higher altitude mountainous area and the central part of the plain area. Construction land covered the smallest area; it was mainly concentrated in the central part of the plain area, with a highly fragmented distribution. Analysis of the structural changes in LULC (Figure 5, Table 2) from 1980 to 2020 showed that the areas of farmland, water areas, and construction land tended to increase, with increases of 2017.90 km^2^, 181.50 km^2^, and 254.27 km^2^, respectively. In particular, the area of construction land increased the most due to population and urbanization changes; it reached 88.16%. The areas of woodland, grassland, and unused land tended to decrease, with decreases of 15.06%, 14.34%, and 4.94%, respectively. The changes in LULC were strongly related to the local social economy, national policies, and agricultural technology development, and various policies and measures may continue to affect the future LULC status in the river basin.

The predicted results showed (Figure 4 and Figure 5 and Table 2) that under the ND scenario from 2020 to 2030, the areas of farmland and grassland will generally remain stable, whereas the areas of water area and construction land will increase by 246.42 km^2^ and 26.24 km^2^, respectively, with percentage increases of 23.49% and 4.84%, respectively. The areas of woodland and unused land will decrease by 22.32 km^2^ and 255.59 km^2^, respectively. Under the EPD scenario, the areas of woodland, grassland, and water area will increase by 37.33 km^2^, 15.87 km^2^, and 237.77 km^2^, respectively, with percentage increases of 8.29%, 0.16%, and 26.10%, respectively. In particular, the trend in the area of woodland will change from decreasing over many years to increasing. The areas of farmland and unused land will decrease by 104.27 km^2^ and 250.40 km^2^, respectively, and thus the main trend will involve conversion from production land to ecological land. Under the EED scenario, the overall area of woodland will remain stable, but the areas of grassland, water area, and construction land will increase by 144.18 km^2^, 252.67 km^2^, and 65.01 km^2^, respectively, with percentage increases of 1.49%, 24.09%, and 11.98%, respectively. The areas of farmland and unused land will decrease by 254.23 km^2^ and 207.80 km^2^, respectively.

### 3.2. Temporal Changes in ESV in the Manas River Basin

**Figure 6:**
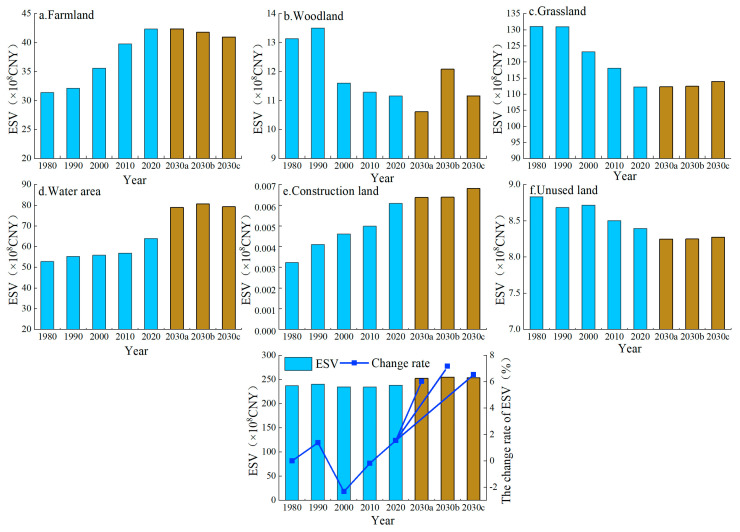
ESVs for LULC types in the Manas River Basin from 1980 to 2030.(Note: a, b, and c indicate ND, EPD, and EED scenarios, respectively).

In this study, a 10-year time scale was used to estimate the changes in the ESV in the Manas River Basin from 1980 to 2020. From 1980 to 2020, the ESV in the study area generally tended to increase from 237.27 × 10^8^ CNY in 1980 to 238.10 × 10^8^ CNY in 2020, an increase of 83.25 × 10^6^ CNY, but the ESV tended to decrease from 1990 to 2010, with the largest decrease of 2.33% between 1990 and 2000, followed by a large increase of 1.54% between 2010 and2020 (Figure 6). Between 2020 and 2030, the ESV in the Manas River Basin will tend to increase under the ND scenario from 238.10 × 10^8^ CNY to 252.47 × 10^8^ CNY, an increase of 14.37 × 10^8^ CNY. Under the EPD scenario, the ESV in the study area will tend to increase significantly to 255.18 × 10^8^ CNY by 2030, with a maximum increase of 7.18%. Under the EED scenario, the increase in the ESV in the Manas River Basin will be lower than that under the EPD scenario, reaching 253.68 × 10^8^ CNY in 2030, with an increase of 6.54%.

Comparisons of the changes in the ESVs for different LULC types (Figure 6) showed that from 1980 to 2020, the areas of farmland, water area, and construction land in the Manas River Basin tended to increase, with the greatest increases for water area and farmland of 11.08 × 10^8^ CNY and 10.95 × 10^8^ CNY, respectively. The ESVs for woodland, grassland, and unused land tended to decrease, with the greatest decrease for grassland of 18.79 × 10^8^ CNY. Comparisons of the average shares of each LULC type in the total ESV in the study area from 1980 to 2020 showed that grassland occupied the largest area with 51.92%, followed by water area and farmland with 24.03% and 15.29%, respectively, whereas the remaining different LULC types had low shares. Under the ND scenario, the ESVs for farmland, grassland, water area, and construction land will all tend to increase from 2020 to 2030, with the greatest increase for water area at 15.02 × 10^8^ CNY, whereas decreases will occur for woodland and unused land. Under the EPD and EED scenarios, the ESVs for woodland, grassland, water area, and construction land will tend to increase, with the most rapid increases for water area in the two scenarios with 26.10% and 24.09%, respectively. In general, the contributions of different LULC types to the ESV in the Manas River Basin followed the order of grassland > water area > farmland > woodland > unused land > construction land.

**Figure 7:**
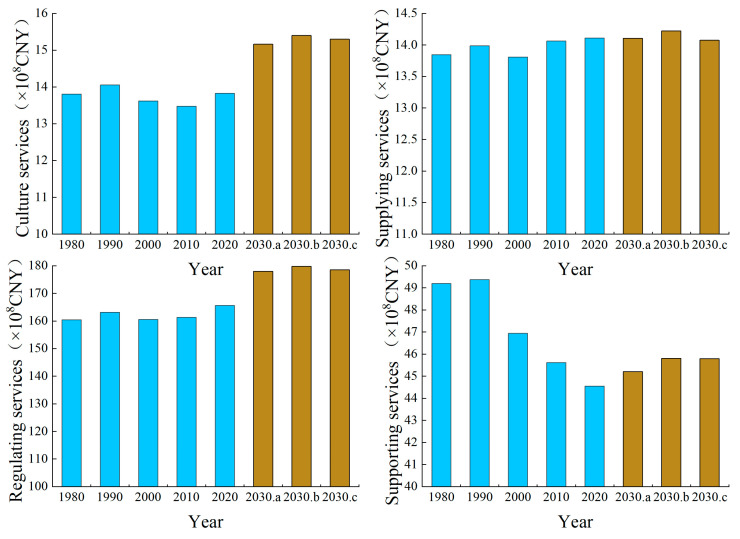
Changes in ESVs for supplying services, regulating services, supporting services, and cultural services from 1980 to 2030. (Note: a, b, and c indicate ND, EPD, and EED scenarios, respectively).

In terms of the primary ecological service functions (Figure 7), the ESVs for the four major service systems in the Manas River Basin from 1980 to 2020 followed the order of regulating services > supporting services > supplying services > cultural services. The ESVs for regulating services, supplying services, and cultural services tended to increase by 3.93%, 1.24%, and 0.16%, respectively, whereas that for support services tended to decrease. By 2030, under the ND and EED scenarios, the ESVs for regulating services, supporting services, and cultural services will all tend to increase, but a slight decrease will occur for supplying services. In particular, the ESV will increase most significantly for regulating services by 12.38 × 10^8^ CNY and 12.90 × 10^8^ CNY under the ND and EED scenarios, respectively. Under the EPD scenario, the ESVs for supply services, regulating services, support services, and cultural services will increase by 11.28 × 10^6^ CNY, 1414.39 × 10^6^ CNY, 125.57 × 10^6^ CNY, and 157.12 × 10^6^ CNY, respectively, mainly due to increases in the areas of ecological land types such as woodland, grassland, and water area under this scenario, with great ecological benefits. In terms of secondary ecosystem service functions (Table 3), gas regulation, climate regulation, water conservation, and waste treatment among regulating services were mainly affected by changes in farmland, woodland, grassland, and water area, but their trends were different. From 1980 to 2030, the ESVs will increase for gas regulation, water conservation, and waste treatment, whereas the ESV for climate regulation will decrease in the Manas River Basin. The ESVs for soil formation and conservation and biodiversity among supporting services will generally tend to decrease in a fluctuating manner, and are related to the reductions in woodland and grassland areas over the years. The food production function among supply services was mainly influenced by changes in the area of farmland, where the value increased due to the continuous expansion of the farmland area. From 1980 to 2030, the value of food production services will increase by 13.67%, 12.96%, and 11.83% under the ND, EPD, and EED scenarios, respectively. Raw material production is greatly affected by woodland and grassland, and its service value will decrease by 10.63%, 8.76%, and 9.05% under the ND, EPD, and EED scenarios, respectively. The ESV due to the provision of aesthetic landscapes among cultural services will tend to increase in a fluctuating manner over the years, but the changes will be small.

### 3.3. Spatial Changes in ESV in the Manas River Basin

**Figure 8:**
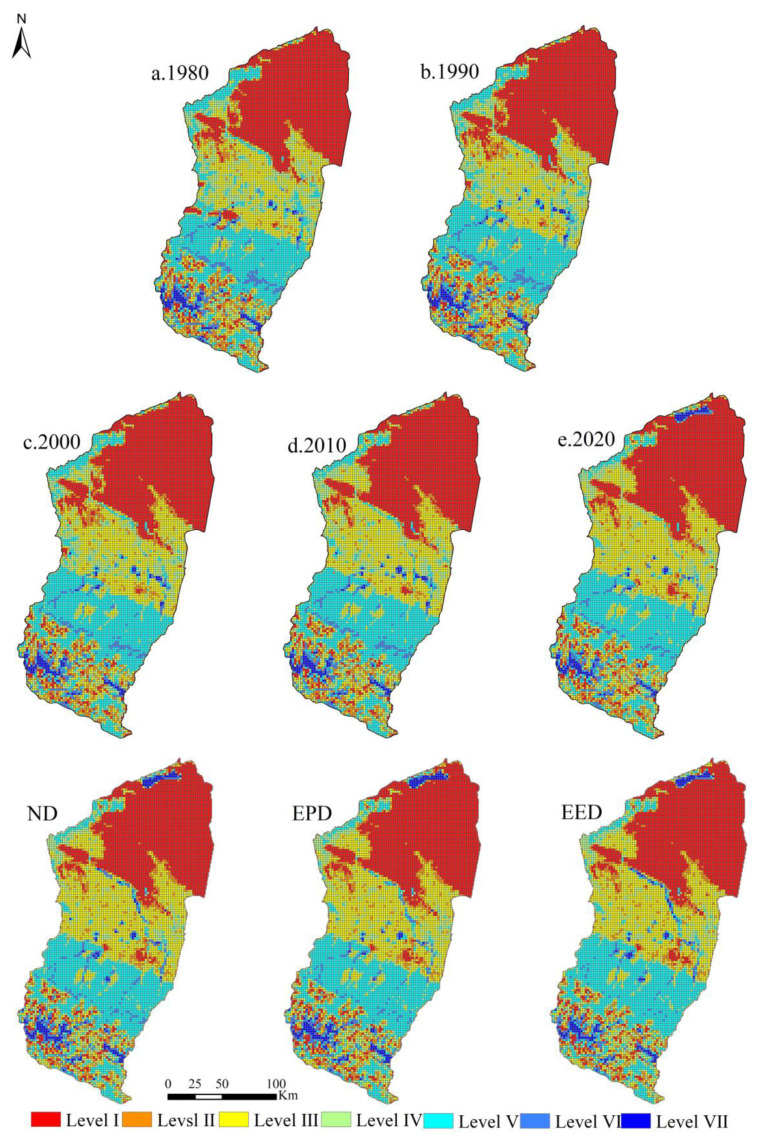
Temporal and spatial variations in the ESV in the Manas River Basin from 1980 to 2030.

The spatial differences determined in the ESV in the Manas River Basin were significant (Figure 8), but the spatial distributions were relatively consistent, with high distributions in the south and low distributions in the north. The areas with high ESV were mainly distributed in the mountainous areas and southern part of the oasis plain area, which are located at high altitude and close to the Tianshan Mountains and have high annual rainfall and snow melt water volumes, large water area, and high woodland and grassland coverage. In addition, human activities and economy had little influence on this area. From the middle part of the oasis plain area to the desert area, the ESV decreased gradually, as the middle part of the basin is a flat plain area rich in water resources with better lighting conditions, concentrated population, and vigorously developing agriculture and economy. The low-ESV areas were mainly distributed in the desert area in the north of the basin, as this area is mainly dominated by desert, and thus it is arid all year with poorly developed vegetation. From 1980 to 2020, the high-ESV area in the Manas River Basin increased gradually and the low-value area decreased continuously. The most significant changes occurred mainly in the oasis area and desert area of Manas Lake, whereas the spatial distribution of the ESV generally remained stable in the southern mountainous and desert areas. The ESV in the Manas River Basin under different scenarios from 2020 to 2030 will tend to increase significantly around the water system but decrease around construction land in the plain area, mainly due to the gradual expansion of the construction land boundary under socio-economic development, thereby resulting in increased pressure on the ecological environment around construction land and decreasing the ESV.

### 3.4. Sensitivity Analysis of the ESV

**Figure 9:**
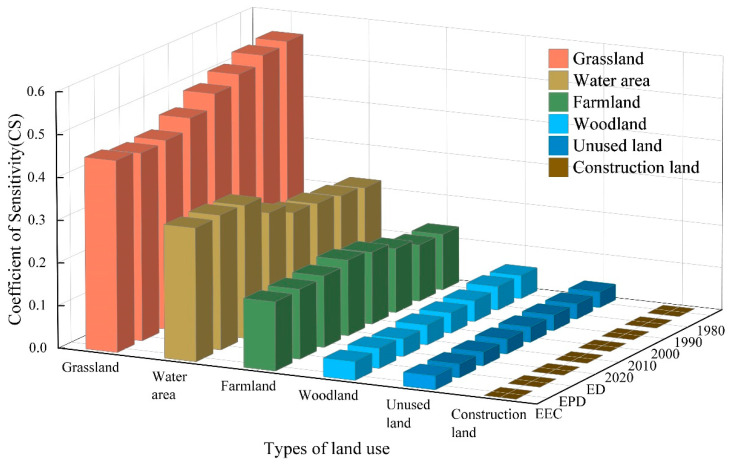
Coefficients of sensitivity for ESV.

The CS values were calculated based on the ESV coefficients for the study area to assess whether the calculated results agreed with the actual situation in the study area. The sensitivity of the adjusted value coefficients was less than one when the ecological value coefficients for LULC types in the Manas River Basin were shifted up and down by 50% to analyze the sensitivity of changes in the ESV to the value coefficients (Figure 9). The sensitivity for different LULC types followed the order of grassland > water area > farmland > woodland > unused land > construction land. Thus, changes in grassland had a greater effect on the ESV in the Manas River Basin, whereas changes in the value of construction land had a smaller effect on the value of ecological services. Sensitivity analysis showed that the basin ESV was not sensitive to the value coefficients used in this study. Thus, the ESV coefficients used in this study agreed well with the actual situation in the Manas River Basin, thereby confirming the validity and credibility of our results.

### 3.5. Ecosystem Service Trade-Offs and Synergies

We explored the trade-offs and synergies for ecosystem services under different scenarios in the Manas River Basin by adjusting the grid scale several times and considering the study area, visualization effects, and research methods. We evaluated the study area under three scenarios in 2030 with a grid unit of 2 km × 2 km, where each grid unit served a single ESV, correlations were calculated, and bivariate global autocorrelation analysis was performed using SPSS. The Pearson correlation coefficient greater than 0 indicates a synergistic relationship between different ecosystem services, and a larger coefficient denotes a more significant synergistic relationship between ecosystem services over time, whereas the opposite indicated a trade-off relationship. The Pearson correlation coefficients and bivariate global autocorrelation Moran’s I indexes calculated for the ND, EPD, and EED scenarios indicated consistent synergistic relationships between supplying services, regulating services, supporting services, and cultural services in the Manas River Basin. The Pearson correlation coefficients and Moran’s I indexes (Table 4) showed that the synergistic relationships were significant for: regulating services—cultural services > supplying services—supporting services > supporting services–cultural services > regulating services–supporting services > supplying services–regulating services > supplying services–cultural services.

**Figure 10:**
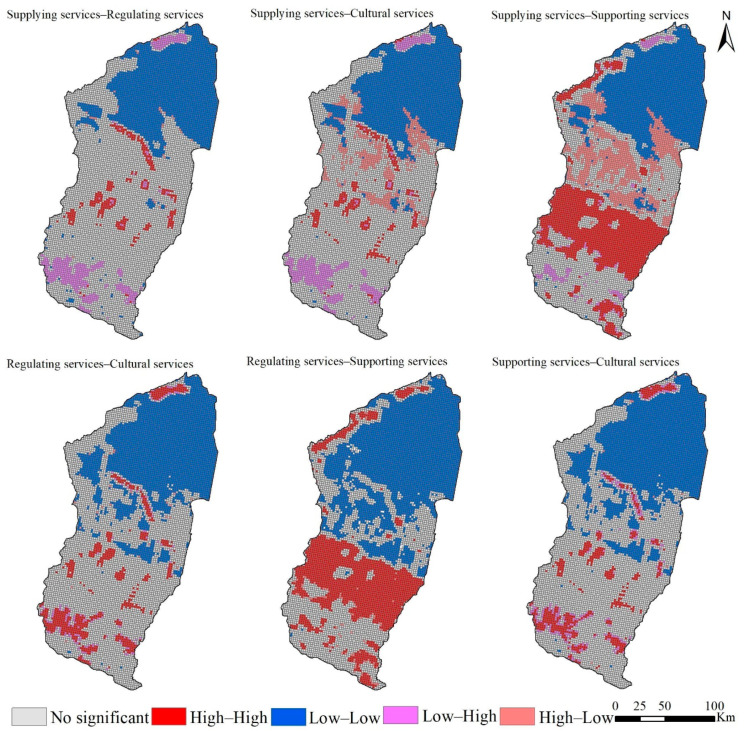
Partial LISA map showing the four ecosystem services in the Manas River Basin under the EED scenario in 2030.

In order to explore the clustering characteristics of the synergistic and trade-off relationships among different ecosystem services in the Manas River Basin, we conducted bivariate local autocorrelation analysis to obtain more detailed results. In recent years, the Manas River Basin pays more attention to the coordinated development of ecology and economy, and considering its significance, the ESV value under the EED scenario was selected to represent the synergy and trade-off relationships among ecosystem services. As shown in Figure 10, the spatial agglomeration characteristics of synergies and trade-offs among ecosystem services were generally significant. Supplying, regulating, supporting, and cultural services mainly had synergistic relationships in space, and they were concentrated in the desert area in the northern part of the Manas River Basin. Local trade-off relationships were mainly distributed in the plain area of the basin. In general, the synergistic relationships in the desert areas in the northern part of the basin were characterized by low–low clustering, mainly due to the effects of the natural geographical characteristics of this region, where desert areas are widely distributed. The synergistic relationships around the water system were characterized by high–high clustering due to the lush vegetation growth and high quality of the ecological environment. The supplying services–regulating services and supplying services–cultural services trade-offs were characterized by high–low clustering in the southern mountainous region. Because the water areas are surrounded by large areas of unused land and the ecological environment is fragile, landscape services were also affected to some extent. Both supplying services–supporting services and regulating services–supporting services had synergistic relationships characterized by high–high aggregation in the mountainous flood plains due to the extensive areas of woodland and grassland and the superior quality of the ecological background; however, supplying services–supporting services had a trade-off relationship characterized by high–low clustering in the plains, mainly due to the conflict between food supply and supporting services as the area of farmland expanded and reductions in the areas of woodland and grassland under economic development. The synergistic relationship between regulating services andsupporting services was characterized by low–low aggregation in the oasis plains, mainly due to the large amount of farmland and the rapid outward urban expansion. The synergies between regulating services and cultural services and supporting services and cultural services were more pronounced in the central–northern part of the plain, mainly because of the extensive farmland in this area and its location on the boundaries of towns, and thus the ecological quality and landscape services were affected to some extent.

## 4. Discussion

The results obtained in the present study demonstrated that changes in LULC could significantly affect the ESV. Using the GMOP–PLUS coupling to optimize the LULC structure addressed the contradiction between the use of land resources and social and economic development in the basin, thereby helping to promote the coordinated development of the ecological environment and social economy. Based on simulations of the LULC in the Manas River Basin in 2030, we estimated that the basin ESV will increase significantly under the three scenarios, where the ESV was found to be largest under the EPD scenario and lowest under the ND scenario. The EED scenario considers both economic and ecological benefits, and the ESV was slightly lower under the EED scenario than that under the EPD scenario; however, this scenario is more suited to the sustainable development of the basin. These findings showed that the GMOP–PLUS model can be used to optimize LULC. Spatial differences in ecosystem services were clearly identified by analyzing the trade-offs and synergies among the ESVs in the basin to facilitate regional LULC planning in a more refined manner.

### 4.1. Response of ESV to Changes in LULC

**Figure 11:**
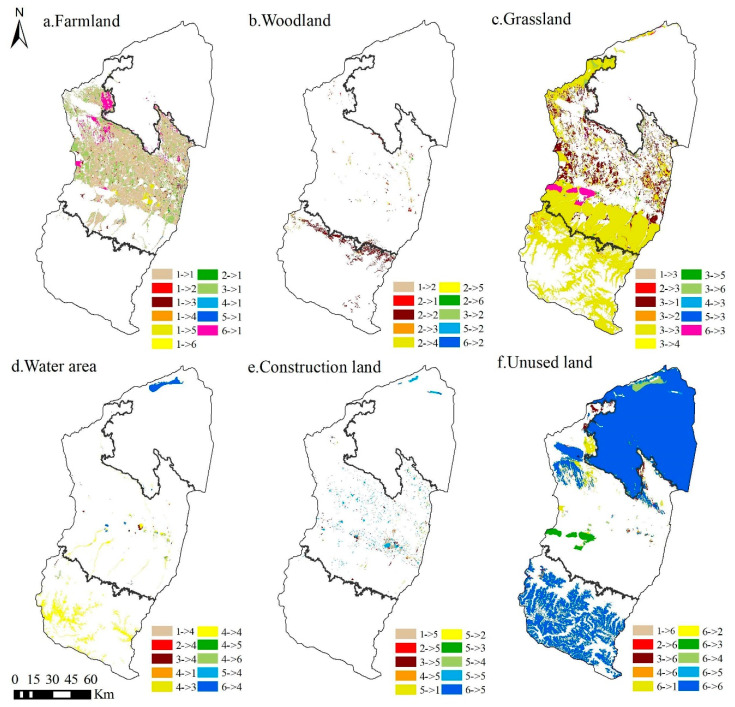
Spatial changes in LULC in the Manas River Basin from 1980 to 2020. (1, Farmland; 2, Woodland; 3, Grassland; 4, Water area; 5, Construction land; 6, Unused land).

In line with previous studies [7,48,55], we found that changes in LULC could significantly affect the ESV. Changes in LULC alter the ability of ecosystems to provide ecosystem goods and services by affecting ecosystem processes and patterns [56]. The degradation of ecosystem services due to rapid urbanization has led to ESV losses [57,58]; however, we found that even in the context of rapid economic and urbanization development, the ESV in the Manas River Basin continued to exhibit small fluctuating increases (Figure 6). This may be explained by the following two reasons: First, the unique physical geography of the Manas River Basin had a positive effect on the stability of the ecosystem; the basin is divided from south to north into mountainous, oasis, and desert areas, and changes in LULC in the mountainous areas are mainly influenced by climate change. As temperatures increase over the years, the areas with snow and ice will gradually decrease, but the overall LULC structure in the mountainous areas will remain stable (Figure 11). The oasis area is the main area for agricultural farming and economic development, and LULC changes are affected significantly by human activities. From 1980 to 2020, the oasis plain area was reclaimed and cultivated on a large scale, and the areas of ecological land such as grassland, water area, woodland, and unused land decreased dramatically, whereas those of agricultural land and land for production and living increased sharply. Therefore, the natural oasis was gradually transformed into an artificial oasis and high-intensity human activities made the ecosystem more fragile. Thus, this was the main area where the ESV decreased. The desert area is dominated by large areas of unused land and the LULC pattern has remained relatively stable over the years (Figure 11). Second, changes in LULC in the Manas River Basin are strongly related to the policies and measures implemented in the region. Since 1980, in the context of reform and opening up, the land policies comprising “household production contracting system” and “household production contracting,” and the land management model for unified and separate management were implemented in the river basin; in addition, due to the development of irrigation technology, the agricultural irrigation mode in the basin changed from flood irrigation to combinations of channels, reservoirs, and well irrigation [59,60]. After 2000, due to the rapid implementation of the “Ninth Five-Year Plan” and “Western Development Policy” in the basin, urbanization and industrialization developed rapidly, and social and economic levels increased, while the number of migrants also increased significantly and the ESV in the study area generally declined. After 2010, due to the introduction of the ecological civilization concept and the ecological protection red line, ecological protection and restoration became of major importance in the basin, and the return of farmland to forest and grassland was actively promoted [61,62]. The ecosystem was restored to some extent and the ecological service capacity and value subsequently increased.

The Manas River Basin is located in the northwest arid region of China, and the amount of water resources is an important factor that affects changes in LULC. Similar to most dry basins, our analyses of the relationships between the amount of water resources, LULC structure, and ESV in terms of geographical characteristics showed that the mountainous areas of the Manas River Basin have high value supporting, regulating, and cultural services due to the abundance of water resources and widespread distributions of woodlands, grasslands, and water area [63,64]. Oasis area mainly relies on water resources formed by mountain runoff to provide ecosystem services [65]; therefore, oasis area in the Manas River Basin has high supplying services value. The oasis zone consumes a large amount of water resources, thereby leading to degradation of the lower part of the basin and the formation of a desert ecosystem as a low-value area for ecological services (Figure 8). The relationships between water resources, LULC, and ESV were also analyzed from the perspective of human activities. After the 1980s, due to the substantial increase in farmland, the water consumed to provide irrigation increased rapidly; the agricultural water consumption accounted for 90% of the total annual water consumption and the surface water utilization rate reached 96% [59]. At the end of the 1980s, groundwater extraction developed from groundwater overflow areas to oasis zones, including from shock flood plains in the upper mountainous regions to the central delta and even extending to the desert areas of the basin [66], thereby overloading the amount of water resources in the basin and leading to rapid reductions in groundwater levels, shrinkage of grassland and water area in the oasis plain areas, and reductions in the value of regulating, supporting, and cultural ecological services, but significantly increasing the value of supplying services. In addition, the construction of massive water conservation facilities in the basin increased the consumption of water resources [63]; the water catchments in the lower reaches of the rivers decreased sharply, thereby making it difficult to guarantee the availability of ecological water, which resulted in declines in woodland and grassland in the lower reaches and decreases in the ESV. After 2000, with the development of water-saving irrigation technology, the utilization degree of water resources in the basin has been continuously improved and agricultural water consumption has been reduced [67]; after 2010, with the proposal of the policy of “returning land and reducing water”, the implementation of “determining land by water and determining crops by water” reduced agricultural water use to compensate for ecological water use and the ESV increased in the basin (Figure 6). Thus, water plays a crucial role in the ecosystem, and changes in the amount of water resources determine the capacity and value of ecosystem services [68,69]. Therefore, the ESV of the water area and its surroundings is high.

### 4.2. LULC Coupled Model at the Basin Scale

At present, socio-economic development and ecological protection are the main contradictory objectives that affect the development of basins, which often include many cities that are undergoing rapid economic growth [70,71]. How to balance the interrelationship between ecological protection and economic growth in a basin and how to achieve high-quality basin development are issues that many countries and regions now need to address. However, coupled models have only been applied previously to ecological functional areas and cities, such as in Wuhan, China [30], the large-scale Sichuan-Yunnan ecological functional area [15], and Atlanta, USA [72], where the LULC structure and ecological and environmental conditions differ from basins. Therefore, it is necessary to explore the application of coupled LULC models at the basin scale by considering the synergistic ecological and economic development of the basin, which is important for achieving high-quality and sustainable development.

LULC change simulation is a complex system process affected by multiple factors. In this study, we combined the GMOP model with the PLUS model and proposed a GMOP–PLUS coupled model, thereby exploiting the structural optimization and spatial allocation of the two models to scientifically construct an LULC structure optimization model for ecosystem research at the basin scale. The GMOP model can solve various uncertainties of objective function and constraint conditions under different scenarios [15], while the PLUS model is widely applicable and not limited to simulations of LULC in urban or natural environments. It enhances the spatio-temporal dynamics in simulations, improves the ability to synchronize the changes in multiple categories and types of patches [30], and it is more suitable for larger scale areas. Land systems are complex systems containing natural, social, and economic components, and LULC optimization aims to maximize the economic, social, and ecological benefits. Considering that social benefits are difficult to quantify, the objective function is established in terms of both economic and ecological benefits. In addition, supplying services, regulating services, and supporting services are basic conditions for the sustainable development of basins. In this study, we prioritized the constraints of ESV from these three aspects, and set the future scenarios of the basin in turn using the GMOP–PLUS coupling model to optimize the LULC structure in future scenarios, analyze the temporal and spatial evolution characteristics of ESV under different scenarios, plan and adjust future LULC structure, and provide decision support for decision makers. We concluded that the coupled model proposed in this study is highly suitable for investigating changes in LULC in basins.

### 4.3. Suggestions for a Basin Development Model Based on the ESV

The LULC pattern in the Manas River Basin has undergone major changes due to rapid urbanization and social economic development. The Manas River Basin remains an underdeveloped region in the west, and economic development is still the main goal of urbanization. Therefore, the reasonable coordination of ecological and economic benefits is a problem that needs to be solved prior to further development of the Manas River Basin. According to previous studies [61,64], the ecosystem structure of the basin is fragile, and the natural background quality is low, while expansion of the oasis area to meet human needs in the basin has become an increasingly prominent issue. We found that the ESV was largest under the EPD scenario because economic benefits were not considered. Under the EED scenario, the ESV was slightly lower compared with that for the EPD scenario due to the full consideration of the economic benefits from urbanization and industrialization, and the policy of “land retreat and water reduction” implemented for ecological protection in recent years. The areas of farmland and construction land tended to decrease and increase, respectively. However, considering the EPD and EED scenarios, the latter might be a better match with the future development model of the basin with increases in construction land. In addition, the “returning farmland to forest,” “returning farmland to grassland,” and “returning land to reduce water” policies have achieved remarkable results, including stabilizing the changes in LULC in the basin, balancing the relationship between land and economy to some extent, and leading to a win–win situation in terms of the development of the basin.

Food security is a fundamental requirement for the development of a basin. Thus, when optimizing the LULC structure, it is necessary to strictly adhere to the red line of farmland protection to ensure that the area of high-quality farmland is not reduced, and this is the basis for the stable ESV under the ND scenario. In addition, the Manas River Basin is constrained by the topographic characteristics of the mountainous and desert areas in the north and south, and the suitable development and construction areas overlap with the areas of high-quality farmland. This is likely to lead to a contradiction between urban infrastructure construction and farmland protection; strict control of the boundaries of urban development and intensive use of land resources are favorable conditions for the realization of the EED scenario. Water resources are an important factor that affects ecological development in the arid regions of Northwest China. In the Manas River Basin, it is necessary to solve the contradiction between providing water resources for agricultural development and compensating for ecological water consumption; thus, low-yield farmland can be transformed into woodland and grassland with higher ecological benefits. In addition, due to the significant increase in the ESV predicted under the EPD scenario in this study, we can explore a reasonable model for transforming a large amount of unused land into ecological land and enhance the planned use of unused land with low ESV in the north to develop its ecological potential. In addition, we can explore the possibility of improving the balance between grassland and livestock by implementing measures such as grazing bans and grazing rotations. In terms of spatial planning, it is necessary to consider the synergistic relationships between the ESV to the north and south of the river basin, as well as strictly implementing the ecological red line policy and protecting the woodland and grassland with high ESV in the mountainous areas and southern plains. According to the “low–low synergy” and “high–low trade-off” relationships between the four services in the central and northern plains of the study area, it is necessary to adjust the agricultural structural and promote ecological restoration and construction. Therefore, it is necessary to scientifically delineate urban development boundaries and basic farmland and ecological protection red lines, prevent the disorderly expansion of construction land, and enhance the service value of the basin ecosystem to allow the unified development of ecological, economic, and social benefits.

### 4.4. Limitations and Future Research

In this study, we applied a new method to optimize the ESV at the basin scale; however, some limitations still need to be addressed to facilitate practical applications of this method. First, we used the value equivalent method to evaluate the ESV and made two revisions based on the food price and biomass. This method is widely accepted and recognized [73,74], but the diversity of ecosystems still leads to differences in the ESV [75]; therefore, it is necessary to revise or establish an indicator system and corresponding value coefficient for the ESV in the study area based on the willingness of local people and their ability to pay in order to make the results more realistic. We will improve this method in future research. Second, it is possible to objectively overestimate or underestimate the future ESV by using the annual value equivalent under current or historical conditions to predict the change in the future ESV. Third, in order to investigate the spatial aggregation pattern in the ESV, we conducted aggregation at a 2-km grid scale to assess synergies and trade-off relationships; however, this type of spatial analysis has complex scale effects [76]. In future research, we can apply the idea of multi-scale spatial changes to compare the ESV, improve the accuracy of LULC data, and explore the impacts of scale effects on the ESV.

## 5. Conclusions

In this study, a coupled GMOP–PLUS model was constructed to optimize the LULC structure and explore the changes in the ESV under different future scenarios, and this method was applied to an inland river basin in the arid area of northwest China. Our main conclusions based on the results obtained in this study are as follows:

(1) The spatial LULC pattern in the Manas River Basin changed significantly. From 1980 to 2020, the areas of farmland, water area, and construction land tended to increase, whereas the areas of woodland, grassland, and unused land tended to decrease. From 2020 to 2030, under the ND scenario, the areas of farmland and grassland will generally remain stable, and the areas of woodland, water area, construction land, and unused land will increase or decrease; under the EPD scenario, a large amount of farmland and unused land will be converted into ecological land, where the area of ecological land will increase by 290.97 km^2^, but the expansion of construction land will be moderate; and under the EED scenario, the areas of grassland, water area, and construction land will tend to increase, where the area of construction land will reach 11.98%.
(2) The ESV in the Manas River Basin showed an increasing trend of fluctuation. From 1980 to 2020, the ESV in the study area increased slightly from 237.27 × 10^8^ CNY to 238.10 × 10^8^ CNY. From 2020 to 2030, the ESV in the Manas River Basin will increase significantly, with increases of 14.37 × 10^8^ CNY, 17.08 × 10^8^ CNY, and 15.58 × 10^8^ CNY under the ND, EPD, and EED scenarios, respectively. The ESV in the Manas River Basin will increase most under the EPD scenario, followed by the EED scenario and then the ND scenario. The spatial differences in the ESV in the Manas River Basin were significant, but the spatial distribution was relatively consistent over the years, with high distributions in the south and low distributions in the north.
(3) From 1980 to 2020, the four individual ESV changes of supplying, regulating, supporting, and culture services in the Manas River Basin are shown as regulating services > supporting services > supplying services > cultural services. From 2020 to 2030, regulating services, supporting services, and cultural services will all tend to increase under the ND, EPD, and EED scenarios, but supplying services will increase or decrease under the three scenarios. From 1980 to 2030, in terms of primary ecosystem services, the ESVs for gas regulation, water conservation, and waste treatment will all tend to increase, whereas those for climate regulation, soil formation and protection, and biodiversity will decrease in a fluctuating manner.
(4) In 2030, the trade-offs and synergies for various ecosystem services will be consistent in the Manas River Basin under the three scenarios with obvious significant synergistic effects, and the significant degree of synergetic relationship will be regulating services–cultural services > supplying services–supporting services >supporting services–cultural services > regulating services–supporting services > supplying services–regulating services > supplying services–cultural services. In terms of the spatial distribution, “low–low synergy” and “high–high synergy” clustering characteristics were obvious in the northern part of the basin and along the water system. Supplying services and the other three services mainly had “high–low trade-off” and “low–high trade-off” relationships in the plains and southern mountainous areas in the middle and north of the basin. The relationships comprising regulating services–supporting cultural, regulating services–cultural services, and supporting services–cultural services in this region were mainly scattered “low-low synergy” relationships, which were affected mainly by the distribution of ecological belts and human activities in the basin.

## Figures and Tables

**Table 1:** Ecological service value equivalents of different LULC in the Manas River Basin (CNY (ha·a)^–1^).

Ecosystem Classification		Farmland	Woodland	Grassland	Water Area	Construction Land	Unused Land
Supplying services	Food production	650.35	112.13	257.9	224.26	0	11.21
	Raw material production	100.92	2915.37	381.24	44.85	0	16.82
Regulating services	Gas conditioning	1244.64	3924.53	1356.77	1009.17	0	11.21
	Climate regulation	571.86	3027.5	3576.93	9844.97	0	78.49
	Water conservation	672.78	3588.14	897.04	20,116.02	0	33.64
	Waste disposal	1838.92	1468.9	1468.9	20,385.13	0	11.21
Supporting services	Soil formation and protection	11.21	4373.05	1513.75	964.31	0	22.43
	Biodiversity	235.47	3655.42	1502.53	2803.24	0	381.24
Cultural Services	Aesthetic landscape	100.92	1435.26	661.56	5550.41	11.21	11.21

**Table 2:** Areas of LULC types in the Manas River Basin from 1980 to 2020, and predictions for 2030 under different scenarios (km^2^).

Year	Farmland	Woodland	Grassland	Water Area	Construction Land	Unused Land
1980	5786.59	536.29	11,281.16	867.08	288.43	15,291.96
1990	5919.35	551.09	11,271.38	907.11	365.42	15,037.16
2000	6557.07	473.64	10,601.43	916.78	410.77	15,091.80
2010	7328.37	460.77	10,162.20	933.06	444.59	14,722.51
2020	7804.49	455.52	9663.77	1048.93	542.70	14,536.10
2030.a	7807.49	433.20	9666.02	1295.35	568.94	14,280.50
2030.b	7700.22	493.29	9679.65	1322.70	569.96	14,285.70
2030.c	7550.26	455.68	9807.96	1301.60	607.71	14,328.29

Note: a, b, and c indicate ND, EPD, and EED scenarios, respectively.

**Table 3:** ESV in the Manas River Basin during 1980–2030 (×10^8^ CNY).

Ecosystem Classification		1980	1990	2000	2010	2020	ND	EPD	EED
Supplying service	Food production	7.10	7.19	7.43	7.81	8.02	8.07	8.02	7.94
	Raw material production	6.74	6.79	6.38	6.25	6.09	6.03	6.21	6.13
Regulating service	Gas conditioning	25.66	25.91	25.50	25.82	25.83	26.00	26.15	25.97
	Climate regulation	55.02	55.48	53.31	52.28	51.88	54.22	54.66	54.72
	Water conservation	33.89	34.82	34.57	34.96	37.14	42.02	42.72	42.18
	Waste disposal	45.85	46.91	47.18	48.27	50.76	55.76	56.23	55.65
Supporting service	Soil formation and protection	20.67	20.75	19.42	18.71	18.05	18.18	18.49	18.50
	Biodiversity	28.53	28.62	27.53	26.91	26.51	27.02	27.31	27.29
Cultural service	Aesthetic landscape	13.80	14.05	13.62	13.47	13.83	15.16	15.40	15.30

**Table 4:** Correlation analysis results for the four ecosystem services under different scenarios in the Manas River Basin in 2030.

Ecosystem Services	Pearson Coefficient			Moran’s I		
	ND	EPD	EED	ND	EPD	EED
Supplying services–Regulating services	0.243	0.256	0.261	0.19	0.206	0.214
Supplying services–Supporting services	0.606	0.597	0.609	0.466	0.468	0.474
Supplying services–Culture services	0.142	0.15	0.157	0.097	0.11	0.119
Regulating services–Supporting services	0.55	0.547	0.551	0.385	0.38	0.38
Regulating services–Culture services	0.987	0.986	0.986	0.637	0.621	0.614
Supporting services–Culture services	0.563	0.562	0.563	0.395	0.394	0.389

## Data Availability

The data presented in this study are available upon request from the corresponding author.
